# A pooled mutational analysis identifies ionizing radiation-associated mutational signatures conserved between mouse and human malignancies

**DOI:** 10.1038/s41598-017-07888-0

**Published:** 2017-08-09

**Authors:** Philip R. Davidson, Amy L. Sherborne, Barry Taylor, Alice O. Nakamura, Jean L. Nakamura

**Affiliations:** 1 Department of Finance and Statistical Analysis, University of Alberta, Edmonton, Alberta T6G 2R3 Canada; 2 Department of Radiation Oncology, University of California, San Francisco, California 94158 USA; 3 Memorial Sloan Kettering Cancer Center, New York, 10065 USA

## Abstract

Single nucleotide variants (SNVs) identified in cancer genomes can be de-convolved using non-negative matrix factorization (NMF) into discrete trinucleotide-based mutational signatures indicative of specific cancer-causing processes. The stability of NMF-generated mutational signatures depends upon the numbers of variants available for analysis. In this work, we sought to assess whether data from well-controlled mouse models can compensate for scarce human data for some cancer types. High quality sequencing data from radiotherapy-induced cancers is particularly scarce and the mutational processes defining ionizing radiation (IR)-induced mutagenesis *in vivo* are poorly defined. Here, we combine sequencing data from mouse models of IR-induced malignancies and human IR-induced malignancies. To determine whether the signatures identified from IR-exposed subjects can be differentiated from other mutagenic signatures, we included data from an ultraviolet radiation (UV)-induced human skin cancer and from a mouse model of urethane-induced cancers. NMF distinguished all three mutagens and in the pooled analysis IR was associated with mutational signatures common to both species. These findings illustrate the utility of pooled analysis of mouse and human sequencing data.

## Introduction

Analyses of thousands of human cancers have revealed cancer genomes to be complex structures bearing the marks of oncogenic germline and somatic alterations, as well as imprints of environmental exposures. The term “mutational signature” refers to the patterns of somatic mutations present in the tumor genome and which serve as a record of the mutational processes leading to cancer development^1^. Characterizations of single nucleotide variants (SNVs) and the local nucleotide contexts in which they arise reveal trinucleotide-based signatures^2^. Cancers harbor multiple trinucleotide-based mutational signatures that can be extracted from whole exome sequencing (WES) data using non-negative matrix factorization (NMF)^3, 4^. Remarkably, different tumor histologies share discrete mutational signatures, with some mutational signatures originating with known mutagenic exposures such as tobacco, ultraviolet light and certain chemotherapies, while others are less understood or are of unknown etiology^3^.

In NMF trinucleotide-based mutational signature analysis, six possible substitutions are considered, based on the pyrimidine in the reference position and the proximal sequence context (one nucleotide 5′ and 3′). SNVs together with their 5′ and 3′ neighboring bases can be organized into 96 trinucleotide-based groups. Large numbers of variants are generally used to identify and distinguish stable trinucleotide-based signatures using NMF. However the extraction of stable NMF signatures may not be possible if the numbers of samples available for study are limited, an issue that is particularly problematic for uncommon clinical samples of limited availability. Attempts have been made to examine the trade-off between number of tissue samples and number of mutations per sample by *Monte Carlo* simulations^2^. This study is, to the best of our knowledge, the first in this area to explore pooling human sequencing data with data from appropriate mouse models to overcome analysis limitations secondary to scarce human cancer samples.

With the exception of common and well-characterized mutagens such as ultraviolet (UV) radiation and tobacco, whose mutational signatures were characterized prior to the development of next generation sequencing^5, 6^, the genome-wide mutational signatures of most mutagens remain uncharacterized. Clinical samples in which to study *in vivo* mutagenesis may, depending upon the mutagen, be limited in availability and arise after unknown and unquantifiable exposures. In contrast to the challenges inherent in studying clinical samples, multiple variables including mutagen exposure can be studied systematically and independently in mouse models, making them powerful tools for studying disease pathogenesis and characterizing mutational mechanisms promoting cancer^4, 7^. Thus, well-annotated cancers from mouse models can potentially inform the analyses of human malignancies, although the utility of this type of cross-species NMF analysis has not been described.

We utilized mouse and human malignancies initiated by ionizing radiation (IR) to study the utility of cross-species analysis. The genetic features of IR-induced malignancies arising *in vivo* are poorly defined. We previously developed a mouse model utilizing anatomically localized (focal) dose-fractionated irradiation, which more accurately models radiation-induced secondary neoplasms in human cancer survivors^8, 9^. Focally irradiated mice develop IR-induced malignancies that resemble clinical IR-induced malignancies both anatomically and histologically^9^. Similar to the clinically-based radiation protocols utilized in our mouse model, the two human IR-induced malignancies included in this analysis were induced by focal fractionated radiation^10^. As a result, the mouse and human IR-induced malignancies shared key similarities in mutagenic exposure with the previously analyzed WES data from the IR-induced malignancies arising within our mouse model^4^.

In prior work we analyzed whole exome sequencing (WES) data from IR-induced malignancies arising within our mouse model^4^. Using this previously described mutation data and WES data from the human IR-induced malignancies, we tested the utility of cross-species analysis by extracting trinucleotide-based mutational signatures from the exomes of IR-induced human tumors as a group and also pooled with WES data generated from a mouse model of IR-induced malignancies.

To test the ability of our procedures to discriminate between different mutagens, we included sequencing data from 22 mouse tumors induced by the chemical mutagen urethane^7^. In addition, two samples were included from a single UV-induced human skin cancer, a known non-IR malignancy control. The trinucleotide mutational signature for UV is defined, thereby providing the opportunity to determine whether our methods and data could reproduce the previously reported UV-signature.

Using WES data for three patients combined with previously published WES data from mouse IR-induced and urethane-induced malignancies, we tested the utility of cross-species analysis by comparing trinucleotide-based mutational signatures from the human tumors as a group with mutational signatures extracted from pooled human and mouse data. Trinucleotide-based signatures from IR, UV or urethane-induced malignancies were robustly extracted by NMF. In addition, the pooled analysis enabled the resolution of an additional IR-related mutational signature that was not detectable prior to pooling but was present in both IR-induced human and IR-induced mouse cancers. This cross-species analysis illustrates an opportunity for using sequencing data from mouse models to uncover insights into human cancer genetics.

## Results

### Normalizing trinucleotide mutational frequencies for NMF analysis

NMF, which utilizes somatic SNVs as input, can be used in the study of cancer genomes to extract patterns reflecting mutagenic processes. However, those patterns may be obscured if NMF is applied to pooled data for which data types harbor inherent differences in trinucleotide frequency (e.g., WGS and WES data).

Similarly, it would make little sense to directly compare trinucleotide-based signatures separately extracted by NMF from datasets of different types for which there are inherent trinucleotide frequency differences.

To illustrate the significance of this issue, we examined previous analyses by Alexandrov *et al*.^3^, and we present two corresponding sets of signatures that differ only in the normalization applied. For the first of these two sets, the signature coefficients were normalized on the basis of the actual trinucleotide frequency of the whole human genome. These signatures are referred to hereafter using the published numbering, as Wellcome Trust Sanger Institute (WTSI) signatures 1A, 1B, 2, …, 21. Updated versions of these 22 WTSI signatures (plus 8 new similarly normalized signatures) are given on the COSMIC site (at http://cancer.sanger.ac.uk/cosmic/signatures), and are referred to hereafter as COSMIC signatures 1–30. (The correlations of WTSI signatures 1A and 1B with COSMIC signature 1 are 1.00 and 0.87, the correlation of WTSI signature 2 with COSMIC signature 2 is 0.85, and the correlations of WTSI signatures 3–21 with COSMIC signatures 3–21, respectively, are all 0.959 or higher; Supplementary Table S1, cols. BC-BX and rows 78–98).

The second set of NMF signatures provided by Alexandrov *et al*.^3^ are normalized according to an equal trinucleotide frequency representation (shown in their Supplementary Fig. S2, panel b - Fig. S23, panel b). They denote these signatures using the same numbering with “(norm)” following each signature number; hence here these signatures are denoted as WTSI signatures n1A, n1B, n2, …, n21. (Each pair of WTSI signatures for the two different normalizations is correlated 0.761 or higher; Supplementary Table S1, columns AG-BB and rows 56–77).

**Figure 1:**
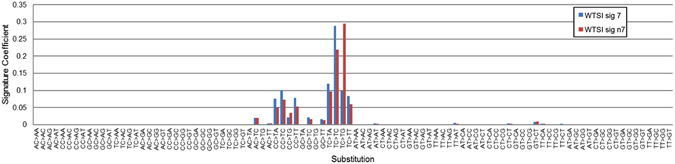
Normalizing trinucleotide frequencies. Figure 1 illustrates the effect of normalization of signatures. UV-associated WTSI signatures 7 and n7 are shown, with blue bars for the first of these signatures, normalized on the basis of the actual trinucleotide frequency of the whole human genome (Alexandrov *et al*.^3^, Supplementary Fig. S9a) and red bars for the second of these signatures, normalized according to an equal trinucleotide frequency representation (Alexandrov *et al*.^3^, Supplementary Fig. S9b). In the case of normalization according to an equal trinucleotide frequency representation, T[C > T]G mutations dominate, whereas in the case of normalization on the basis of the actual trinucleotide frequency of the whole human genome, T[C > T]C mutations dominate.

Alternative normalizations of NMF signatures can influence the composition of trinucleotide signatures. Consider the UV-associated WTSI signatures 7 and n7 shown in Fig. 1, with blue bars for the first of these signatures, normalized on the basis of the actual trinucleotide frequency of the whole human genome (Alexandrov *et al*.^3^, Supplementary Fig. S9a) and red bars for the second of these signatures, normalized according to an equal trinucleotide frequency representation (Alexandrov *et al*.^3^, Supplementary Fig. S9b). In the case of normalization according to an equal trinucleotide frequency representation, T[C > T]G mutations dominate, whereas in the case of normalization on the basis of the actual trinucleotide frequency of the whole human genome, T[C > T]C mutations dominate. This comparison illustrates that normalization choices influence signatures even when the NMF signatures are derived from data for the same species (e.g., all human data in the case of the WTSI signatures 7 and n7).

To address known human-mouse differences in trinucleotide frequencies, in this work we perform normalization based on an equal trinucleotide frequency representation. We expect that conserved human-mouse mutational patterns associated with specific mutagens will be more evident when using a normalization that equalizes the probability of a purely random mutation at any one trinucleotide.

### NMF signatures identified in normalized human-only mutational frequency data

In the human-only NMF analysis, we included tumor samples initiated by either IR or UV. Two IR-induced malignancies from patients were analyzed by whole exome sequencing (a single tumor sample from one patient, Patient 1, and 2 anatomically separate tissue samples of the same tumor from the other, Patient 2), revealing a total of 194 single nucleotide variants (SNVs) for Patient 1, and a total of 361 SNVs for Patient 2 (202 in sample A and 159 in sample B)^10^. The third clinical case was a UV-associated squamous cell carcinoma of the scalp (Patient 3). For Patient 3, two tumor samples were taken at one year apart (designated as pre- and post-) and were known not to have received radiotherapy. All tumor samples were analyzed and compared to the matched germline. Each of Patient 3’s tumor samples possessed significantly more SNVs than those of either Patient 1 or 2 (1861 in the pre- sample and 1978 in the post- sample, for mutation loads of 29.2 and 31.0 mutations per Mbp, respectively). Prior descriptions of UV-associated skin cancers indicate that these malignancies typically bear higher mutational loads as compared to other, non-UV associated malignancies^3^. The mutational loads occurring in our skin cancer samples compare similarly to previously published rates in human skin cancers.

**Figure 2:**
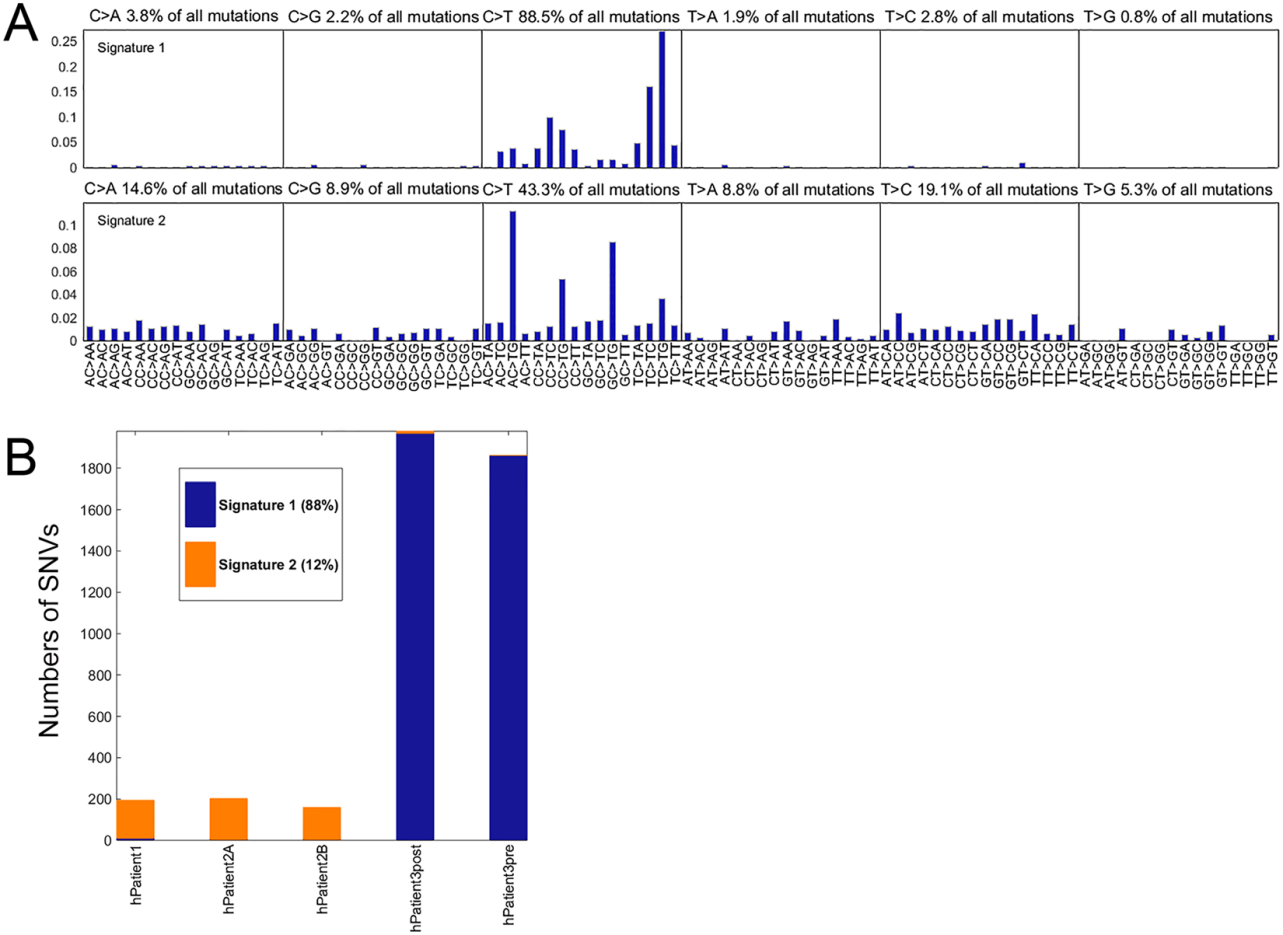
Mutational signatures in human malignancies induced by IR or UV. (**A**) Two discrete mutational signatures were identified in 5 tissue samples from 3 human patients, one (Patient 3) with skin cancer and two (Patient 1 and 2) with tumors induced by previous radiation therapy. Normalization was performed before NMF. The plots show the distribution of the six mutation types defined by the pyrimidine base in each signature, as inferred from the NMF procedure. Each sub-graph within a signature represents one substitution (e.g., C → A when **C** in the reference genome is mutated to A in the sample). The bars within each sub-graph include the nucleotides in the reference genome on either side of the mutation location (e.g., AC > AG represents A at 5′, C in the reference mutated to A, and G at 3′), 96 substitution types shown. Almost all the mutations in Signature 1 are C → T substitutions (88.5%) but the weights differ markedly by neighboring nucleotides (e.g. TC > TG is much more prevalent than AC > TG). Signature 2 has more C → T substitutions than any other type (43.3% of all mutations in signature 2), but differs from Signature 1 substantially in neighboring nucleotides (AC > TG here is much more prevalent than TC > TG). (**B**) Signature 1 is defined by the skin cancer exomes while signature 2 is defined by IR-induced malignancies.

Performing NMF analysis as previously described^2, 4^, the normalized mutational frequency data from the somatic SNVs in the five human tumor samples for the three patients (the P123 dataset) were found to support the extraction of two stable signatures (Fig. 2A and Supplementary Fig. S1). Signature 1 is almost exclusively in the skin cancer samples for Patient 3, while signature 2 is almost exclusively in the IR-induced samples for Patients 1 and 2 (Fig. 2B). The mutations assigned to the two signatures significantly differ between the skin cancer and the IR-induced cancer samples (p-value 1.5 × 10^−173^ for a likelihood ratio test).

### Distinguishing mutational signatures in IR, UV and urethane-induced human and mouse cancers

Analyzing the UV-induced human tumor exomes with IR-induced human tumor exomes demonstrated that two distinct mutational signatures could be distinguished in a small cohort of samples (Fig. 2). In assessing the value of introducing the exomes of mouse malignancies to further resolve mutational signatures, we included IR-induced mouse malignancies and a previously described non-IR control mouse model of chemical tumorigenesis^7^ to test the robustness of our approach. This alternative dataset introduced an additional mutational signature found in mouse non-small cell lung cancers resulting from isolated exposure to urethane^7^.

**Figure 3:**
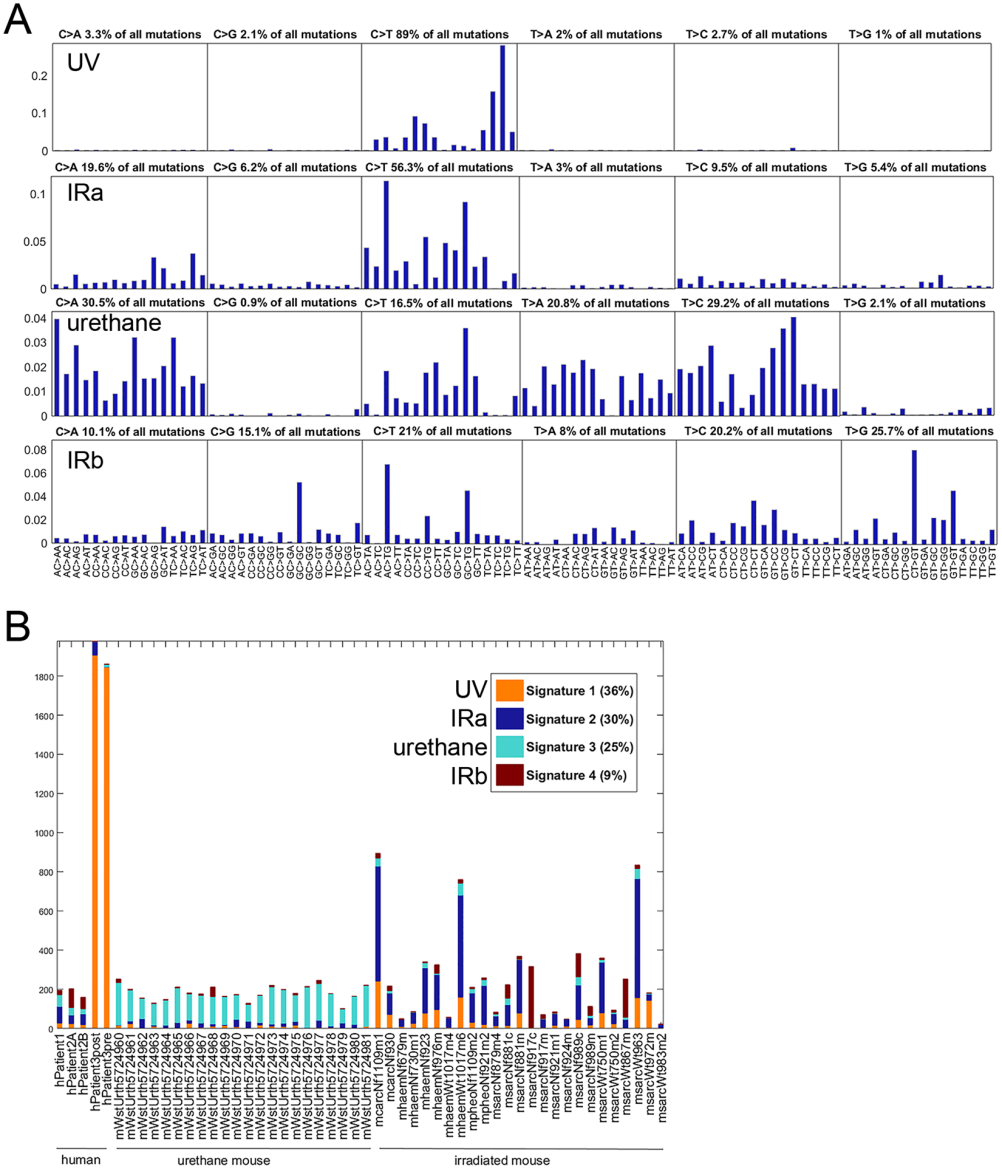
Mutational signatures in human and mouse malignancies induced by IR, UV or chemical mutagenesis. (**A**) The coefficients of 4 signatures from the 52 samples represented in (**A** and **B**). The majority (56.6%) of mutations represented by Signature 2 are C → T, but as seen in the first analysis, the neighboring bases differ markedly from the neighboring bases contributing to Signature 1, suggesting that the likelihood of C → T mutations to UV versus IR depends on the trinucleotide context. Signatures 3 and 4 are more complex than either of the others. Signature 3 has more C → A (31.2%) than any other type, with T → C, T → A and C → T all fairly prevalent. Signature 4 has approximately equal numbers of C → T, T → C and T → G mutations. (**B**) Shown are the number and proportion of mutations in each signature for each tissue sample when the 5 human tumors from the previous analysis are augmented with data from 25 IR-induced mouse samples and 22 urethane-induced mouse tumors. Signature 1 (UV) is still defined by the 2 samples from the skin cancer patient. However, adding the mouse tumors allows 2 IR signatures and one chemical signature (urethane, Signature 3) to be differentiated.

We pooled the normalized data for Patients 1, 2 and 3 (the P123 dataset) with the normalized data for the IR-induced mouse malignancies (the IRmse dataset), and with normalized data for 22 samples from urethane-induced mouse malignancies (the Uremse dataset; derived from a file included in Westcott *et al*.)^7^. NMF analysis of this pooled P123+IRmse+Uremse dataset permitted the extraction of four stable signatures, shown in Fig. 3A. Extraction of four signatures (Supplementary Fig. S2) is supported by the finding that successively increasing the number of signatures extracted from one to four resulted in successive significant decreases in the residual NMF error (p-values < 0.0001) whereas increasing signature extraction from four to five signatures did not produce a significant reduction in the residual NMF error (p-value = 0.48). In addition, the stability metric for the solution fell from more than 0.9 at four signatures to 0.6 at five signatures and dropped further for greater numbers of signatures (Supplementary Fig. S2).

All four of the NMF signatures extracted from the P123+IRmse+Uremse dataset (shown in Fig. 3A, ordered there as they were extracted by NMF; coefficients given in Supplementary Table S2, cols. Q-T) can be interpreted based on enrichment in samples from tumors experimentally induced by IR or urethane exposures or by conformity with WTSI signature n7 that was previously determined to reflect UV-related mutagenesis. Moreover, these four signatures are not highly correlated with each other (Table 1, rows 1–4), suggesting that they represent separate mutational processes. To our knowledge, this is the first NMF cancer study where all the identified signatures can be associated with well-defined mutagens.

**Table 1:** Correlations of signature coefficients.

Row	The human or human-mouse dataset from which the signature(s) were extracted, and the number of the signature, based on the order of the signatures extracted by NMF/the total number of NMF signatures extracted	P123+IRmse+Uremse 1/4 (UV signature)	P123+IRmse+Uremse 2/4 (IRa signature)	P123+IRmse+Uremse 4/4 (IRb signature)	P123+IRmse+Uremse 3/4 (urethane signature)
Correlations among the four signatures extracted from the P123+IRmse+Uremse dataset					
1.	P123+IRmse+Uremse 1/4 (UV signature)	1			
2.	P123+IRmse+Uremse 2/4 (IRa signature)	0.144	1		
3.	P123+IRmse+Uremse 4/4 (IRb signature)	−0.020	0.369	1	
4.	P123+IRmse+Uremse 3/4 (urethane signature)	−0.114	0.238	0.027	1
Correlations with the normalized WTSI signatures correlated most highly with the UV, IRa, IRb and urethane signatures					
5.	WTSI sig n7	0.970	0.039	−0.082	−0.146
6.	WTSI sig n6	0.335	0.853	0.463	0.229
7.	WTSI sig n14	0.185	0.816	0.421	0.282
8.	WTSI sig n17	0.165	0.020	0.698	−0.120
9.	WTSI sig n8	0.169	0.539	0.323	0.421
10.	WTSI sig n5	0.266	0.576	0.261	0.416
Correlations with signatures extracted from alternative datasets					
14.	P3 1/1	**0.999**	0.153	−0.014	−0.109
15.	P123 1/2	**0.999**	0.151	−0.015	−0.110
16.	P123 2/2	0.314	**0.795**	0.521	0.270
17.	P123+IRmse 1/3	**1.000**	0.151	−0.014	−0.109
18.	P123+IRmse 2/3	0.192	**0.996**	0.349	0.275
19.	P123+IRmse 3/3	−0.013	0.483	**0.974**	0.138
20.	P12+IRmse 1/2	**0.649**	**0.842**	0.250	0.149
21.	P12+IRmse 2/2	0.019	0.534	**0.955**	0.168

We pooled the normalized data for Patients 1, 2 and 3 (the P123 dataset) with the normalized data for the IR-induced mouse malignancies (the IRmse dataset), and with normalized data for 22 samples from urethane-induced mouse malignancies. Correlations with the normalized WTSI signatures correlated most highly with the UV, IRa, IRb and urethane signatures are shown in rows 5–10.

Signature UV (Fig. 3A), which is enriched in the skin cancer samples, visually resembles the Signature UV of the human tumors-only analysis (Signature 1 in Fig. 2A) and the two signatures are almost perfectly correlated (correlation 0.999, row 15, Table 1). Among the compatibly normalized WTSI signatures, our UV signature is most highly correlated with WTSI Signature n7, which represents UV (correlation 0.97 in row 5, Table 1).

Two distinct IR signatures can now be differentiated. The single IR-associated Signature 2 extracted from the human-only P123 dataset now fractionates here into signatures IRa and IRb. These signatures are not highly correlated (correlation 0.369, row 3 Table 1), suggesting they represent separate biological processes. Among the compatibly normalized WTSI signatures, the IRa signature is most highly correlated with WTSI signatures n6 and n14 (correlations of 0.853 and 0.816, rows 6 and 7, Table 1), and is also quite highly correlated with WTSI signatures n1A, n1B, n15, and n19 (correlations of 0.764, 0.770, 0.677 and 0.714; Supplementary Table S1, col. P). The IRb signature is most highly correlated with WTSI signature 17n (correlation of 0.698, row 8, Table 1), with that being the only correlation over 0.60 for IRb with any of the compatibly normalized WTSI signatures (Supplementary Table S1, col. R).

Signature 3 of the four signatures extracted from the P123+IRmse+Uremse dataset is predominant in the samples for the urethane-treated mice (Fig. 3B), and hence is referred to hereafter as the urethane signature. The highest correlations of this signature with any of the compatibly normalized WTSI signatures are 0.421 and 0.416 for WTSI signatures n8 and n5 (Supplementary Table S1, col. Q), indicating that the urethane signature is not represented (or is not accurately represented) in any of the mutational signatures described to-date for human malignancies.

The UV, IRa, IRb, and urethane signatures all have substantial representation of C → T substitutions. C → T substitutions predominate for both the UV signature (89%) and the IRa signature (56.3%), though with different nucleotide contexts. The IRb signature has more T → G (25.7%) than C → T (21%) substitutions. The urethane signature has more C → A substitutions than any other type (30.5%), followed by T → C (29.2%) substitutions.

### Comparing the IRa, IRb and UV signature results with radiation-associated signatures extracted from mouse IR-induced malignancies

In prior work, IR-associated signatures were identified using mouse data for 192-mutational types (i.e., taking account of the strand in that prior work along with the 96 trinucleotide-based mutation types considered here to facilitate comparisons with the WTSI and COSMIC signatures)^4^, and without normalization. Differences between the signatures obtained in that prior study and those identified here are potentially due to the use of mouse-only versus pooled mouse-human data, the use of un-normalized versus normalized data, and the number of mutational types considered.

Rows 1–5 in Table 2 shows pair-wise correlations between the UV, IRa, IRb, urethane signatures and new signatures from the normalized IRmse dataset (which does not include human data and no data from the urethane-treated mice). This dataset is the normalized, 96-mutational type counterpart of the un-normalized, 192-mutational type dataset used in our *Cell Reports* (CellR) paper^4^. Rows 1–2 give correlations when two signatures are extracted, and rows 3–5 give correlations when three signatures are extracted from the IRmse dataset.

**Table 2:** Correlations of the four signatures from the P123+IRmse+Uremse dataset with signatures from only the irradiated mouse samples, IRmse, with and without normalizing frequencies before NMF.

Row	The human or human-mouse dataset from which the signature(s) were extracted, and the number of the signature, based on the order of the signatures extracted by NMF/the total number of NMF signatures extracted	The signatures extracted from the pooled P123+IRmse+Uremse dataset after normalizing and shown in Fig. 3A:			
		P123+IRmse+Uremse 1/4 (UV signature)	P123+IRmse+Uremse 2/4 (IRa signature)	P123+IRmse+Uremse 4/4 (IRb signature)	P123+IRmse+Uremse 3/4 (urethane signature)
1.	IRmse 1/2	0.642	0.848	0.255	0.152
2.	IRmse 2/2	−0.023	0.382	0.981	0.083
Correlations with the 96 trinucleotide-type, normalized counterpart of the *Cell Reports* (CellR) 3 signatures for mouse-only data					
3.	IRmse 1/3	0.436	**0.921**	0.299	0.240
4.	IRmse 2/3	**0.779**	0.521	0.151	−0.017
5.	IRmse 3/3	−0.033	0.299	**0.980**	0.042
Correlations with the 96 trinucleotide-type, un-normalized counterpart of the 192 trinucleotide-type *Cell Reports* (CellR) 3 signatures for mouse data					
6.	CellR 1/3	**0.530**	0.266	−0.004	−0.071
7.	CellR 2/3	0.305	**0.688**	0.060	0.172
8.	CellR 3/3	−0.095	−0.013	**0.840**	−0.046

Table 2 shows the correlations for (1) four signatures extracted from the pooled P123+IRmse+Uremse dataset shown in Fig. 3A, with normalized NMF input frequencies, (2) three signatures extracted from the IRmse dataset only, with normalized NMF input frequencies, and (3) three signatures extracted from the IRmse dataset, but with un-normalized frequencies. Of the three NMF-derived signatures for the normalized IRmse data, the first of the three signatures is correlated 0.92 with the IRa signature, and the third of these signatures is correlated 0.98 with the IRb signature.

In Table 2, the IRa signature has a correlation of 0.921 with signature 1/3 (row 3), the IRb signature has a correlation of 0.980 with signature 3/3 (row 5), and the UV signature has a correlation of 0.779 with signature 2/3 (row 4). That third correlation is higher than expected given that none of the mouse IR-induced malignancies were skin cancers and these cancers arose in mice that were not exposed to UV. However, it supports the possibility that one of the three previously reported IR-related mutational signatures^4^ represents a process also operating in cancers induced by UV radiation. This possibility is consistent with the statistically significant correlation of 0.649 (p-value < 0.0001) between signature 1/2 extracted from the P12+IRmse dataset (containing no data from the skin cancer of Patient 3), and the UV signature (Table 1, row 20).

The low correlations of the urethane signature with signatures 1/3, 2/3 and 3/3 (rows 3–5 in Table 2: correlations of 0.240, −0.017, and 0.042 further supported by the Table 1, the row 4 low correlations of the urethane signature with the UV, IRa and IRb signatures: −0.114, 0.238 and 0.027) suggest that urethane-mediated mutagenesis involves different mechanisms.

Finally, in Table 2, rows 6–8, correlations are shown for the UV, IRa, IRb and urethane signatures with three signatures extracted from the un-normalized 96-type WES data for the IR-exposed mice (the CellR dataset hereafter, which is the un-normalized counterpart of the IRmse dataset). The highest correlation in each of the first three columns of rows 6–8 is considerably lower than the corresponding highest correlation in rows 3–5. This is to be expected since the correlations in rows 3–5 are between signatures extracted from compatibly normalized datasets, whereas this is not the case for the correlations in rows 6–8.

### Presence of IRa and IRb signatures in IR-associated human cancers examined in another study

In this analysis we sought to also determine whether the IR signatures we identified were present in the sequencing data of other known IR-associated human malignancies to date. Behjati *et al*.^11^ reported the mutational analysis of 12 radiation-associated second malignancies arising in human cancer survivors. Prominent somatic mutations (not derived from NMF) consisted of deletions and balanced inversions, as opposed to the SNVs that are our focus. To examine this independent dataset for the presence of the IR-associated signatures identified above, we pooled the SNV data for the 12 IR-induced cancers examined in the Behjati *et al*. study (included as a supplementary file in that work^11^), suitably normalized and referred to hereafter as the IRB dataset, with our normalized data for Patients 1, 2 and 3 and for the IR-exposed mice. The pooled P123+IRmse+IRB12 dataset allows extraction of seven signatures before the stability falls steeply.

Table 3 shows the correlations of the four signatures (rows 1–4) and the seven signatures (rows 5–11) extracted from this alternative dataset with the UV, IRa, IRb and urethane signatures previously defined. The UV signature is correlated 0.997 and 0.998, with signatures 1/4 and 1/7 (col. 1 of Table 3). The IRa signature is correlated 0.922 and 0.987 with signatures 2/4 and 2/7 (col. 2 of Table 3). The IRb signature is correlated 0.603 and 0.784 with signatures 3/4 and 5/7 (col. 3 of Table 3). The correlations for the urethane signature are all less than 0.5 (col. 4, Table 3). These correlations and the NMF exposure results (not shown) suggest that the UV, IRa and IRb signatures are present in the IRB samples, but the urethane signature is not.

**Table 3:** Effect of including WTSI irradiated samples.

Signatures extracted from the P123+IRmse+IRB dataset	The signatures extracted from the pooled P123+IRmse+Uremse dataset after normalizing and shown in Fig. 3A:			
	P123+IRmse+Uremse 1/4 (UV signature)	P123+IRmse+Uremse 2/4 (IRa signature)	P123+IRmse+Uremse 4/4 (IRb signature)	P123+IRmse+Uremse 3/4 (urethane signature)
1. P123+IRmse+IRB 1/4	**0.997**	0.149	−0.025	−0.115
2. P123+IRmse+IRB 2/4	0.329	**0.922**	0.434	0.259
3. P123+IRmse+IRB 3/4	−0.051	0.150	**0.603**	0.062
4. P123+IRmse+IRB 4/4	0.121	0.591	0.242	0.497
5. P123+IRmse+IRB 1/7	**0.998**	0.139	−0.027	−0.116
6. P123+IRmse+IRB 2/7	0.174	**0.987**	0.351	0.240
7. P123+IRmse+IRB 3/7	0.331	0.809	0.460	0.212
8. P123+IRmse+IRB 4/7	0.076	0.543	0.264	0.466
9. P123+IRmse+IRB 5/7	−0.051	0.060	**0.784**	−0.111
10. P123+IRmse+IRB 6/7	0.206	0.457	0.187	0.329
11. P123+IRmse+IRB 7/7	0.132	0.145	0.049	0.241

Correlations of signature coefficients from P123+IRmse+Uremse 4-signature analysis with each other and the sample including the 12 WTSI irradiated samples, P123+IRmse+IRB.

## Discussion

Analyses of thousands of diverse human malignancies have shown that cancer genomes reflect multiple influences and pathogenetic events^3, 4, 12^. The challenge, then, is to de-convolve the totality of somatic abnormalities present in cancer genomes into interpretable motifs that provide insights into cancer origin and evolution. As a statistical approach, NMF is sensitive to variant numbers and large numbers of variants are generally used to obtain mutational signatures with high stability^2^. However, apart from common cancers and the most common environmental exposures, the mutational imprints produced by many known and suspected carcinogens remain poorly identified.

As a result, most mutational signatures described to date are produced by poorly defined mechanisms as compared to specific, well-characterized mutagens such as ultraviolet radiation and tobacco that produce characteristic mutational signatures. IR is one such mutagen whose trinucleotide-based mutational signature has only recently been elucidated^4^.

Human cancers arise in diverse individuals after variable and often undocumented exposures, which complicates efforts to analyze discrete mechanisms of genotoxicity and cancer promotion. Furthermore, in certain patient populations germline variants are increasingly detected and recognized as facilitating tumor formation, which further complicates tumor analyses depending on the genetic variant under study. When attempting to isolate mutational motifs related to discrete genetic events, analyzing clinical samples may be limiting. To create datasets of sufficient size to extract stable, replicable NMF signatures and for carrying out other sorts of statistical analysis where the size of the dataset can matter, researchers sometimes pool samples from subjects reported to have been exposed to the same mutagen (e.g., IR), but lacking relevant specifics of those exposures and details regarding other potentially confounding factors.

Mouse models are invaluable *in vivo* experimental systems in which to study cancer pathogenesis and mutational mechanisms underlying human cancers^7^. Mouse models have multiple specific advantages for complementing clinical samples and can overcome some of the limitations faced when clinical samples alone are analyzed by sequencing. These advantages include the homogeneity of the genetic background and the ability to control environmental exposures precisely.

This work demonstrates that exomic data from well-controlled experimental systems can be pooled with human data to facilitate insights into the mutational profiles of human cancers. NMF, when based on mutation frequency data normalized according to the appropriate reference genome trinucleotide frequencies, represents the mutation incidence so that the probability of a purely random mutation at any one trinucleotide is equalized. This work reports for the first time that the same trinucleotide-based mutational signatures characterize both IR-induced human malignancies and IR-induced mouse malignancies. These findings argue in favor of mouse models as relevant tools with which to study mechanisms of human cancer pathogenesis.

These findings also suggest that mutation spectra are not necessarily exclusive. The UV and urethane-induced malignancies harbored single signatures with little mixing of additional signatures. In contrast, the known IR-induced malignancies from both humans and mice harbored signatures shared with the known UV and urethane-induced malignancies. Because neither the human and mouse IR-induced malignancies were exposed to UV or urethane the presence of what we identified as the UV and urethane signatures in these malignancies as well suggests that IR or other mutational processes not yet identified can recapitulate these signatures.

Ionizing radiation interacts directly with DNA, producing double-strand breaks, but also produces chemical changes in DNA through the production of water-derived free radicals also known as reactive oxygen species^13^. As secondary events, reactive oxygen species interact with DNA through a variety of mechanisms, producing base damage, crosslinking and single strand breaks^13–15^. The relative diversity of free radicals and molecular interactions induced by IR may help to explain the complexity of IR-associated mutational signatures, why multiple signatures are present in IR-induced malignancies, and why some of these signatures may be present in malignancies produced by other non-IR mutagens. This is an important area for further research.

In contrast to IR, the UV signature, which was extracted from only two tissue samples from one patient, closely matched the signature previously established^3^. This would indicate that a large number of mutations from a very small number of samples can yield reliable signatures, a finding that holds particular significance for mutational analyses of data from small numbers of highly mutated clinical samples.

In this work, an unsupervised analysis demonstrates that multiple mutagenic processes can be simultaneously distinguished in human and mouse cancers. Previously reported IR-related trinucleotide signatures were found in IR-induced human malignancies, using the same NMF extraction methods and normalized mutational input data. This is an important step forward, though much future work remains to improve the precision of the estimated signatures coefficients and to explore their generality.

The deconstructSigs method^16^ utilizes previously identified mutational signatures previously identified in comprehensive analyses of human cancers, and presents an approach that uses this information to enable mutational signature analysis of clinical samples on an individual basis. This method determines the contributions of each of the specified previously identified mutational signatures to a single tumor sample^16^. Operationally, this methodology involves an *a priori* assumption of the mutational signatures expected to be present. In contrast, we show an unsupervised analysis and demonstrate that multiple mutagenic processes can be simultaneously distinguished in human and mouse cancers. Our findings suggest that the deconstructSigs approach could use results from well-controlled mouse models where human data are scarce.

Cancer mouse models demonstrating fidelity to human cancers enable detailed studies of pathogenesis^17, 18^. Our findings suggest that other mouse models of cancer formation or mutagenesis may similarly be used to extract and clarify mutational motifs where limited numbers of human samples are available. Experimental mouse models may also be particularly effective for de-convolving, or extracting, mutational signatures when cancers arise from exposures that are imprecisely defined, as is frequently the case for human malignancies.

## Methods

### Human Research Protection

All work was performed under a research protocol approved by the UCSF Committee on Human Research (IRB protocol 11–07304). All work was performed in accordance with relevant guidelines and regulations. Informed consent was obtained from all subjects.

### Sample Preparations

Genomic DNA was isolated from fresh frozen tumor samples and fresh peripheral blood samples using previously described techniques^4^.

### Whole exome sequencing

Whole exome sequencing was performed using the NimbleGen Human exome v3.0 kit. Captured material was indexed and sequenced on the Illumina GAII and HiSeq. 2000 platform at the Institute for Human Genetics at UCSF. Successfully sequenced reads were then mapped to the human reference genome (GRCh37) using GATK best practices and MuTect was used for somatic mutation detection^19^. All tumor samples were analyzed and compared to the matched germline.

### Normalization

Differences in the trinucleotide frequencies between species can influence the relative weighting of SNVs. Hence either separate sets of NMF signatures must be extracted from single-species datasets with the resulting NMF signature coefficients then being compatibly normalized before clustering or other analyses, or the mutational data for each of the species must be comparably normalized prior pooling or other analysis of the NMF results. In this work, all datasets were normalized prior to application of NMF (except as indicated). Exome regions in the reference genomes, GRCh37 (hg19) for humans and NCBIM37 (mm9) for mice, were obtained from bed files downloaded from the Agilent web site (http://www.agilent.com). Trinucleotide frequencies were calculated by Python code. Supplementary Table S3 gives the normalized mutation frequencies for Patients 1, 2 and 3. Supplementary Table S4 gives the sources for all previously published data utilized in this work.

### Data Analysis

Non-negative matrix factorization (NMF) was performed following protocols developed by WTSI^2^.

### Data Availability Statement

References with locations for previously published data (WTSI, human IR, mouse IR and mouse urethane datasets) are provided in the body of the manuscript when the datasets are introduced and discussed. Patient 3’s data is available from the corresponding author.

## Electronic supplementary material

Supplementary Information

Supplementary Tables
