# Supplementary material for: A pooled mutational analysis identifies ionizing radiation-associated mutational signatures conserved between mouse and human malignancies
Source: Sci Rep. 2017 Aug 9;7:7645. doi: 10.1038/s41598-017-07888-0 (PMC5550450; doi:10.1038/s41598-017-07888-0)
Supplement: Supplementary file 1 — Supplementary Information [file 41598_2017_7888_MOESM1_ESM.pdf]

SUPPLEMENTARY MATERIALS

A pooled mutational analysis identifies ionizing radiation-associated mutational signatures conserved between mouse and human malignancies

Philip R. Davidson[1], Amy L. Sherborne[2], Barry Taylor[3], Alice O. Nakamura[1], Jean L. Nakamura[2]

[1]Department of Finance and Statistical Analysis
University of Alberta, Edmonton, Alberta. T6G 2R3 Canada

[2]Department of Radiation Oncology
University of California, San Francisco, California. 94158 USA

[3]Memorial Sloan Kettering Cancer Center
New York, New York. 10065 USA

SUPPLEMENTARY FIGURE

SUPPLEMENTARY FIGURE S1

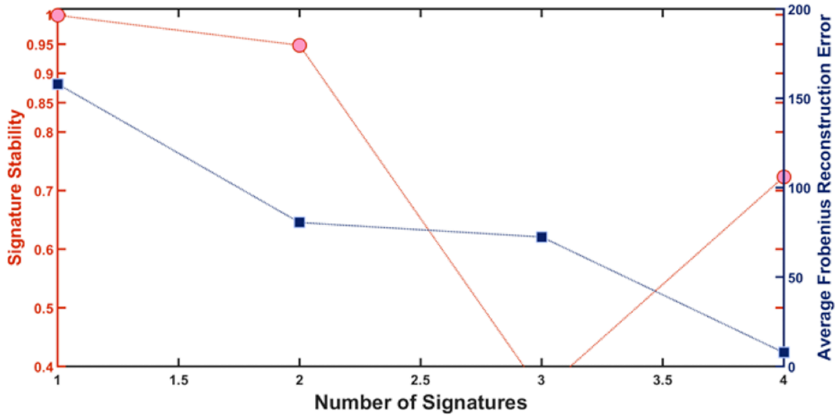

Supplementary Figure S1. Signature stability and reconstruction error

Signature stability and the average Frobenius reconstruction error are shown. Five samples from 3 human patients show signature stability falling dramatically after 2 signatures are extracted. No more than 2 signatures can be extracted reliably with only 5 samples.

SUPPLEMENTARY FIGURE S2

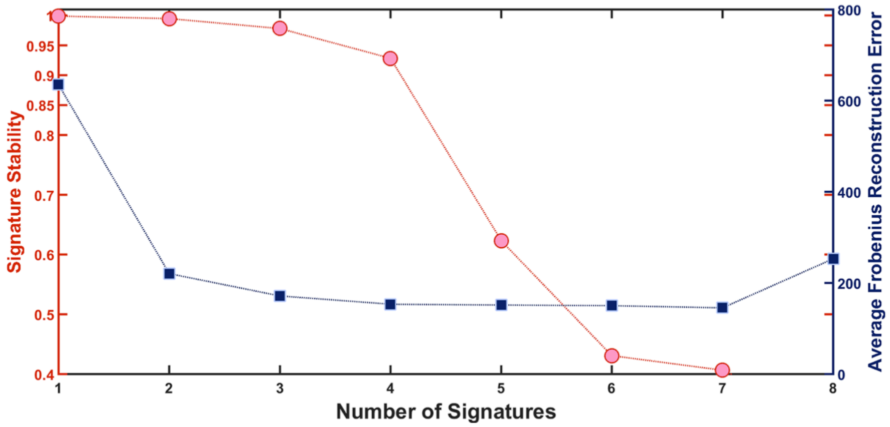

Supplementary Figure S2. **Signature stability and reconstruction error for the pooled human and mouse analysis.**

Signature stability and the average Frobenius reconstruction error are shown for the signature analysis involving the human and mouse malignancies induced by IR, UV or chemical mutagenesis. Extraction of four signatures is supported by the finding that successively increasing the number of signatures extracted from one to four resulted in successive significant decreases in the residual NMF error (p-values < 0.0001) whereas increasing signature extraction from four to five signatures did not produce a significant reduction in the residual NMF error (p-value = 0.48). In addition, the stability metric for the solution fell from more than 0.9 at four signatures to 0.6 at five signatures and dropped further for greater numbers of signatures.

SUPPLEMENTARY TABLE LEGEND

Supplementary Table S1: Correlations of NMF signatures

The S1 sheet of the supplementary data depository Excel file includes correlation coefficients among all signatures extracted in analyses reported in this paper.

Supplementary Table S2: NMF signature coefficients

The S2 sheet of the supplementary data depository Excel file includes the signature coefficients referenced in the paper. All signatures except those labeled CellR were derived from normalized mutational frequency data. Those labeled CellR were derived using un-normalized frequency data (in Table S4).

Supplementary Table S3: The P123 data

The S3 sheet of the supplementary data depository Excel file includes the normalized mutation frequencies for Patients 1, 2 and 3 used in NMF analyses reported in this paper that involve the P123 dataset.

Supplementary Table S4: Consolidated sources for previously published NMF signatures and datasets, and attributes of datasets utilized

The S4 sheet of the supplementary data depository Excel file includes the sources for all previously published NMF signatures as well as for all previously published datasets used in this study (as the source of each was noted when first introduced), and also summarizes relevant attributes of the various datasets used in this study.
